# Sleep quality, physical activity, and perceived academic performance of Italian adolescents

**DOI:** 10.3389/fpubh.2026.1810897

**Published:** 2026-05-13

**Authors:** Alexandro Andrade, Thais Cristina Siqueira, Anderson D’Oliveira, Verônica Maria Claudino, Lavinia Falese, Stefania Mancone, Pierluigi Diotaiuti, Luca Stabile, Giorgio Buonanno

**Affiliations:** 1 Health and Sports Science Center – CEFID/Santa Catarina State University – UDESC, Florianopolis, SC, Brazil; 2 Laboratory of Sports and Exercise Psychology – LAPE, Florianopolis, SC, Brazil; 3 Department of Human Sciences, Society and Health/University of Cassino and Southern Lazio, Cassino, FR, Italy; 4 Department of Civil and Mechanical Engineering, University of Cassino and Southern Lazio, Cassino, FR, Italy

**Keywords:** adolescent health, mental health, psychological well-being, school performance, students

## Abstract

**Background:**

Sleep disturbances are highly prevalent during adolescence and may negatively affect health and academic functioning. During the COVID-19 pandemic, changes in daily routines and lifestyle behaviors may have further impacted sleep patterns and physical activity levels in this population. Although associations between physical activity, sleep, and academic performance have been previously explored, evidence remains limited in specific sociocultural contexts, such as among Italian adolescents during the pandemic. The present study aimed to evaluate sleep quality using the Pittsburgh Sleep Quality Index (PSQI) and to examine its associations with self-perceived academic performance and weekly time dedicated to physical activity among Italian adolescents during the COVID-19 pandemic.

**Methods:**

This cross-sectional study was conducted between May and July 2020 with adolescent students (12–20 years) from public and private schools in the Lazio region, Italy, recruited through convenience sampling. Data were collected via a self-administered online questionnaire (Google Forms). Sleep quality was assessed using the Pittsburgh Sleep Quality Index (PSQI, Italian version). Physical activity and academic performance were evaluated through self-reported measures, including weekly activity duration (minutes/week) and perceived school performance. Descriptive statistics were calculated, and inferential analyses included chi-square or Fisher’s exact tests, Pearson or Spearman correlations, and binary logistic regression models controlling for sex and age to estimate odds ratios (95% CI). Statistical significance was set at *p* < 0.05. The study was approved by an ethics committee.

**Results:**

A total of 437 adolescents (50.8% girls), with a mean age of 15.9 ± 1.37 years, were included. Based on the PSQI, 64.5% of participants presented poor sleep quality. Mean physical activity was 276.6 ± 217.6 min/week, and 67.9% were classified as physically active. Most adolescents reported good or excellent academic performance. Weekly physical activity time was negatively correlated with PSQI total score (*r* = −0.104; *p* = 0.04) and sleep latency (*r* = −0.102; *p* = 0.03). In the adjusted logistic regression model, physical activity was not significantly associated with sleep quality (OR 1.49; 95% CI 0.95–2.33; *p* = 0.079), while sex remained significantly associated.

**Conclusion:**

Italian adolescents presented a high prevalence of poor sleep quality during the COVID-19 pandemic. Although physical activity showed weak correlations with sleep parameters, it was not independently associated with sleep quality after adjustment. These findings highlight the importance of considering potential confounding factors when examining sleep-related behaviors in adolescents. Public policies that focus on sleep quality for adolescents are recommended, since it is possible to observe that several factors can affect adolescent sleep.

## 1 Introduction

Sleep is a fundamental component of adolescent health, a developmental stage characterized by profound biological, emotional, and cognitive changes (1). Adequate sleep plays a critical role in emotional regulation, psychological well-being, and cognitive functioning, whereas insufficient or poor-quality sleep has been consistently associated with adverse physical, mental, and academic outcomes in adolescents (2, 3). During this period, individuals are particularly vulnerable to sleep disturbances due to biological changes in sleep physiology, especially circadian phase delay, which shifts sleep onset and wake times to later hours and often conflicts with early school schedules (4, 5). Although the National Sleep Foundation recommends 8–10 h of sleep for adolescents aged 14–17 years, many fail to meet these recommendations regularly (6).

Sleep problems are highly prevalent during adolescence and arise from the interaction between biological vulnerability and psychosocial and behavioral demands, including academic pressure, stress, and increased screen exposure (7–10). These disturbances may negatively impact daytime functioning, emotional regulation, and school-related outcomes, reinforcing the relevance of sleep as a central component of adolescent health and development (10, 11).

Because the present study was conducted between May and July 2020, the COVID-19 pandemic context is particularly relevant for interpreting the findings. During this period, adolescents experienced substantial disruptions in daily routines, including school closures, changes in academic activities, reduced opportunities for physical activity and sport participation, increased sedentary behavior, and altered sleep schedules (12–17). These changes have been shown to affect both movement-related behaviors and sleep patterns, suggesting that these domains may be closely interconnected, particularly under conditions of social restriction and routine disruption (18, 19).

Movement-related behaviors are important determinants of adolescent health and should be conceptually distinguished (20, 21). Physical activity refers to any bodily movement produced by skeletal muscles that results in energy expenditure, whereas physical exercise is a structured and planned subset of physical activity (22, 23). In contrast, physical inactivity refers to insufficient levels of physical activity, while sedentary behavior is characterized by low-energy expenditure activities performed in a sitting or reclining posture (21, 24). Within this framework, structured exercise and sport participation have been described as potentially protective behaviors for both physical and mental health, with possible benefits for sleep-related outcomes (25). However, adolescents represent a population with high levels of physical inactivity and sedentary behavior, particularly in school and screen-based contexts (21, 26, 27).

A growing body of evidence demonstrates that movement behaviors are associated with brain health and cognitive functioning in youth (28). Previous studies indicate that physical activity contributes to improvements in attention, executive function, and overall cognitive performance (29, 30). These effects are thought to occur through neurocognitive pathways, particularly involving executive functions such as working memory, inhibitory control, and cognitive flexibility, which are closely linked to academic achievement (31). These findings suggest that movement behaviors may influence academic outcomes through neurocognitive pathways, reinforcing their relevance in the educational context (32). Global estimates indicate that a substantial proportion of adolescents do not meet recommended physical activity levels, with more than 80% classified as insufficiently active worldwide (33).

Although previous studies have examined the independent associations between physical activity, sleep, and academic outcomes, recent evidence highlights the importance of considering these behaviors within an integrated 24-h movement framework, given their interdependent nature (34). However, studies simultaneously examining these variables in specific sociocultural contexts, such as during the COVID-19 pandemic, remain limited. In particular, little attention has been paid to the role of general physical activity in relation to sleep quality and academic performance. This gap is especially relevant in the context of the COVID-19 pandemic, during which behavioral patterns were markedly altered, and within specific cultural contexts that remain underrepresented in the literature.

Therefore, the present study aimed to evaluate sleep quality, assessed using the Pittsburgh Sleep Quality Index (PSQI), and to examine its association with self-perceived academic performance and the amount of physical activity performed per week (expressed in weekly minutes) among Italian adolescents during the COVID-19 pandemic period.

## 2 Materials and methods

### 2.1 Study design

This was a descriptive cross-sectional study with a quantitative approach conducted between May and July 2020, using self-administered online questionnaires. The study took place during the COVID-19 pandemic in Italy, a period characterized by partial social restrictions and modifications in school routines. During data collection, schools were operating under adapted conditions, including remote or hybrid learning formats, rather than full closure or strict home confinement.

These contextual conditions may have influenced adolescents’ daily behaviors, including sleep patterns and opportunities for different physical activity practices, due to changes in routine and increased screen time. Therefore, the COVID-19 context was considered a relevant environmental factor for interpreting the findings.

From an analytical perspective, the pandemic context was not included as an independent variable, as all participants were exposed to similar public health measures during the same period. Instead, it was treated as a contextual factor and considered in the interpretation of the results.

The following variables were collected and categorized: (1) sociodemographic variables (age, sex, school year, city of residence); (2) health-related variables (self-perceived health, smoking, medication use, anthropometric data); (3) behavioral variables (weekly time dedicated to physical activity); and (4) outcome variables, including sleep quality assessed by the Pittsburgh Sleep Quality Index (PSQI) and self-perceived academic performance.

### 2.2 Participants

The study population consisted of adolescent students from the Lazio region of Italy. A convenience sampling strategy was adopted due to logistical constraints during the COVID-19 pandemic. Schools were initially contacted via email and/or institutional communication channels to present the study objectives and procedures. Recruitment was conducted through school administrators and teachers, who disseminated the study invitation and the survey link to students. Participation was voluntary, and students accessed the questionnaire through an online platform (Google Forms). If the student had any questions while completing the questionnaire, they could contact a researcher who was ready to answer their questions via email. The contact email was provided in the research description before the student began completing the questionnaire.

Due to the remote recruitment process, it was not possible to precisely determine the total number of schools contacted or the response rate at the institutional level. However, all participants were recruited within the same geographic region and during the same time frame, which may partially reduce variability related to contextual factors. Nevertheless, the use of convenience sampling and voluntary participation may have introduced selection bias, and this limitation should be considered when interpreting the findings.

The inclusion criteria were students of both sexes, aged between 12 and 20 years, regularly enrolled in schools in the Lazio region. Although individuals aged 20 years may be classified as young adults, they were included to represent late adolescence, a transitional developmental stage commonly addressed in epidemiological studies.

Exclusion criteria included self-reported physical or mental conditions that limited motor, sensory, or cognitive abilities, contraindications to physical activity, and incomplete questionnaire responses. These conditions were assessed based on self-reported information, without clinical verification, which may have introduced potential misclassification bias.

No *a priori* sample size calculation was performed. However, considering an expected prevalence of poor sleep quality of approximately 50% in adolescents, a minimum sample of around 384 participants would be required to estimate prevalence with a 95% confidence level and a 5% margin of error. The final sample (*n* = 437) exceeded this threshold, supporting the adequacy of the sample size for descriptive and exploratory analyses.

### 2.3 Procedures

Data collection took place between May and July 2020. Initially, prior contact was made with the schools to publicize the research and explain the research objectives and procedures. Links to access the electronic form in the Google Forms application were made available to students via messaging applications or email. Participants, along with their guardians, were instructed to read and electronically sign the Informed Consent Form (ICF). The ICF detailed the study’s objective, informed students and guardians that participation was voluntary, and reaffirmed their right to withdraw from the study at any time. After electronically signing the ICF, students were instructed to complete the questionnaires.

After participants had completed and signed the informed consent form, data collection began using an identification form prepared by the researchers to collect data on age, sex, city of residence, school year, weight, and height, as well as on the students’ perception of their academic performance, health status, and physical activity (in and outside of physical education at school). In a second stage of data collection, participants responded to the Pittsburgh Sleep Quality Index (PSQI; Italian version) adapted to the digital environment, which aimed to investigate the sleep quality of the participants.

- The Pittsburgh Sleep Quality Index (PSQI), Italian version, was used to assess sleep quality. The PSQI was originally developed by Buysse et al. (42) and later validated for the Italian population by Curcio et al. (43), demonstrating adequate reliability and internal consistency. This is a self-administered questionnaire that assesses sleep quality and patterns through specific questions about the previous month. The results are grouped into seven components, such as subjective quality, latency, duration, usual efficiency, disorders, medication use, and daytime dysfunctions. The results are evaluated on a scale of 0 to 3, with 3 being the negative end of the scale. The values of the domains are summed to obtain an overall score ranging from 0 to 21, with higher scores indicating poorer sleep quality. The scale is categorized into good sleep quality (score 0–5 points) or poor sleep quality (score >5). The PSQI presents high internal consistency (Cronbach’s *α* reliability coefficient = 0.83) and high test–retest reliability, with a product–moment correlation of Pearson’s global score between T1 and T2 of 0.85 (*p* < 0.001) 20.
- Academic performance was assessed through a single self-reported question asking participants to rate their overall school performance. Responses were categorized into three groups: good/excellent, normal, and mediocre/insufficient. Although this measure was subjective and not based on a validated academic scale, self-perceived academic performance has been widely used in adolescent health research as an indicator of school-related functioning.
- Physical activity was assessed through self-report using the question: “How many hours per week do you dedicate to exercise and sport?” Participants were asked to report the total amount of physical activity per week. Total weekly minutes were calculated. Participants were classified as physically active (≥150 min/week) or insufficiently active (<150 min/week), according to World Health Organization recommendations. However, it should be noted that this measure did not distinguish activity intensity, and therefore may not precisely reflect moderate-to-vigorous physical activity (MVPA) as defined by WHO guidelines.

### 2.4 Data analysis and processing

Data were collected using Google Forms, exported to Microsoft Excel, and analyzed using the SPSS. Descriptive statistics were calculated to characterize the sample. Continuous variables were expressed as means and standard deviations, and categorical variables as absolute and relative frequencies. The primary outcome of the study was sleep quality, assessed using the Pittsburgh Sleep Quality Index (PSQI), analyzed both as a continuous variable (total score) and as a categorical variable (good vs. poor sleep quality). Secondary outcomes included specific PSQI domains, such as sleep latency and sleep efficiency. The main independent variables were weekly physical activity (minutes/week) and self-reported academic performance. Sex was included as a covariate in the analyses due to its potential influence on sleep patterns and health behaviors.

Data distribution was evaluated using the Kolmogorov–Smirnov and Shapiro–Wilk tests, complemented by visual inspection of histograms and Q–Q plots. Associations between categorical variables were assessed using chi-square or Fisher’s exact tests, as appropriate. Correlations between continuous variables were analyzed using Pearson or Spearman coefficients, depending on the distribution.

Binary logistic regression models were performed to examine the association between physical activity (150 vs. < 150 min/week) and sleep quality (good vs. poor). Physical activity was analyzed both as a categorical variable (≥150 vs. <150 min/week) and as a continuous variable (minutes/week). The continuous model was considered the primary analysis to explore dose–response relationships, while the categorical model was used for comparison based on public health recommendations. The models were adjusted for sex and age, and results were reported as odds ratios (OR) with 95% confidence intervals (CI). Exploratory analyses using physical activity quartiles were also conducted to evaluate potential non-linear associations with sleep efficiency.

To ensure model robustness, multicollinearity was assessed using variance inflation factors (VIF) and tolerance values. Linearity in the logit for continuous variables was verified via the Box–Tidwell procedure. Specifically, the interaction term between physical activity and its natural logarithm was non-significant (*p* = 0.571), confirming the assumption of linearity. Model fit was evaluated using the Hosmer–Lemeshow test, while explanatory power was determined by Nagelkerke’s *R*^2^. Standardized residuals were examined to identify potential outliers.

To control for multiple comparisons, the false discovery rate (FDR) was applied using the Benjamini–Hochberg procedure. All previously significant associations remained statistically significant after adjustment. Other figures and tables were presented in the Supplementary material.

## 3 Results

The sample consisted of 437 students, with a mean age of 15.97 ± 1.37 years, predominantly female (50.8%), of Italian nationality (98.2%). The mean weight of the participants was 62.7 (±13.2) kg, and the mean height was 170 (±8.37) cm.

Table 1 presents the characterization of the sample regarding Body Mass Index (BMI), perception of health quality, frequency of smoking and getting sick, use of medications, perception of sleep quality, self-reported academic performance, and time spent in physical activity.

**Table 1:** Sociodemographic, health, physical activity characteristics, and division into active and insufficiently active groups of adolescents.

Variables	Teenagers (*n* = 437)	≥150 min/week of reported physical activity (*n* = 297)	<150 min/week of reported physical activity (*n* = 140)
Age (mean ± SD)	15.97 ± 1.37	15.95 ± 1.37	16.03 ± 1.3
Weight (mean ± SD)	62.7 ± 13.2	63.0 ± 13.0	62.2 ± 13.6
Height (mean ± SD)	170.36 ± 8.37	171.00 ± 8.53	169.06 ± 7.87
IMC	*N (%)*	*N (%)*	*N (%)*
- Low weight	60 (13.7%)	45 (15.2%)	15 (10.7%)
- Normal Weight	320 (73.2%)	215 (72.4%)	105 (75.0%)
- Overweight	38 (8.7%)	26 (8.8%)	12 (8.6%)
- Obesity grade I	6 (1.4%)	5 (1.7%)	1 (0.7%)
- Obesity grade II	1 (0.2%)	0 (0.0%)	1 (0.7%)
- Extreme obesity	12 (2.7%)	6 (2.0%)	6 (4.3%)
Sex	*N (%)*	*N (%)*	*N (%)*
- Female	222 (50.8%)	147 (49.5%)	75 (53.6%)
- Male	215 (49.2%)	150 (50.5%)	65 (46.4%)
Perception of health quality	*N (%)*	*N (%)*	*N (%)*
- Good/very good	*319 (73.0%)*	154 (51.8%)	62 (44.3%)
- Normal	*114 (26.1%)*	92 (31.0%)	39 (27.9%)
- Weak/bad	*4 (0.9%)*	51 (17.2%)	39 (27.8%)
Frequency of getting sick	*N (%)*	*N (%)*	*N (%)*
- Rarely/never	300 (68.7%)	208 (70%)	92 (65.8%)
- Sometimes	117 (26.8%)	76 (25.6%)	41 (29.3%)
- Often	20 (4.6%)	13 (4.4%)	7 (5%)
How often do you smoke	*N (%)*	*N (%)*	*N (%)*
- Rarely/never	386 (88.3%)	263 (88.5%)	123 (87.9%)
- Sometimes	22 (5.0%)	12 (4%)	10 (7.1%)
- Often	16 (3.7%)	15 (5.1%)	1 (0.7%)
- Ever	13 (3.0%)	7 (2.4%)	6 (4.3%)
Continuous medication use	*N (%)*	N (%)	N (%)
- Yes	44 (10.1%)	30 (10.1%)	14 (10%)
- No	393 (89.9%)	267 (89.9%)	126 (90%)
Perception of sleep quality	*N (%)*	N (%)	N (%)
- Good/excellent	157 (36%)	118 (39.7%)	39 (27.9%)
- Normal	150 (34.3%)	100 (33.7%)	50 (35.7%)
- Bad/very bad	130 (29.7%)	79 (26.6%)	51 (36.4%)
Perception of academic performance			
- Good/excellent	203 (46.5%)	139 (46.8%)	64 (45.7%)
- Normal	193 (44.2%)	132 (44.4%)	61 (43.6%)
- Mediocre/insufficient	41 (9.4%)	26 (8.7%)	15 (10.7%)
Minutes per week of physical activity (mean ± SD)	276.6 ± 217.6	370.65 ± 204.23	79 ± 43.91
Do you attend physical education classes?	*N (%)*	*N (%)*	*N (%)*
- Yes	428 (97.9%)	294 (99%)	134 (95.7%)
- No	9 (2.1%)	3 (1%)	6 (4.3%)

SD, standard deviation; F, absolute frequency; %, relative frequency/percentage.

Regarding physical activity participation, 97.9% of students reported participating in physical education classes. The reported weekly average time dedicated to physical activity, including physical education classes and activities performed outside school, was 276.6 (±217.6) min. For analytical purposes, weekly physical activity time was examined both as a continuous variable and by quartiles.

Sleep quality was initially assessed by students’ self-report through a specific question in the characterization questionnaire. When asked “How would you rate the quality of your sleep in the last few days?,” the majority of students (70.6%) reported good to normal sleep quality. However, when considering the total score on the specific PSQI questionnaire, it was observed that 64.5% of students presented poor sleep quality, while 35.5% presented good sleep quality. This suggests that the adolescents’ subjective perception of sleep quality does not correspond to their reality during this period. Table 2 presents the characteristics of the sleep quality index according to the PSQI domains.

**Table 2:** Frequency of sleep quality questionnaire domains.

PSQI Index	*N* (%)
*Subjective quality*	
Very good	23 (5.3%)
Good	103 (23.6%)
Bad	254 (58.1%)
Too bad	57 (13%)
*Sleep latency*	
<= 15 min	173 (39.6%)
16 to 30 min	143 (32.7%)
31 to 60 min	67 (15.3%)
>60 min	54 (12.4%)
*Sleep duration*	
>7 h	346 (79.2%)
6 to 7 h	50 (11.4%)
5 to 6 h	24 (5.5%)
<5 h	12 (2.8%)
They did not answer	5 (1.1)
*Sleep efficiency*	
>85%	272 (62.2%)
75 to 84%	69 (15.8%)
65 to 74%	23 (5.3%)
<65%	22 (5.0%)
They did not answer	51 (11.7%)
*Sleep disorders*	
Not once	43 (9.8%)
Less than 1 time/week	332 (76.0%)
1 to 2 times/week	53 (12.1%)
3 times/week or more	9 (2.1%)
*Sleeping medications*	
Not once	416 (95.2%)
Less than 1 time/week	12 (2.7%)
1 to 2 times/week	6 (1.4%)
3 times/week or more	3 (0.7%)
*Daytime dysfunctions*	
None	154 (35.2%)
Less than 1 time/week – small	215 (49.2%)
1 to 2 times/week – moderate	54 (12.4%)
3 times/week or more – A lot	14 (3.2%)
*PSQI – total*	
Good sleep quality	155 (35.5%)
Poor sleep quality	282 (64.5%)

*F* = absolute frequency; % = relative frequency/percentage.

Inferential analyses were performed to verify the presence of associations between the variables related to sleep quality, and the level of physical activity, self-reported academic performance, and sex, as presented in Table 3.

**Table 3:** Associations between PSQI domains and physical activity practice, academic performance, and sex.

PSQI and domains	Physical activity		*p*-value
	Insufficiently active	Active	
*PSQI total*			0.03^*^
Good sleep quality	41(26.5%)	114(73.5%)	
Poor sleep quality	99(35.1%)	183(64.9%)	

PSQI and domains	Academic performance		*p*-value
	Insufficiently/mediocre	Normal/excellent	
*PSQI – subjective*			0.02^*^
Good/very good	18(43.9%)	108(27.3%)	
Bad/Very bad	23(56.1%)	288(72.7%)	
*PSQI – disorders*			<0.001^*^
1x/week or less	25(61%)	350(88.4%)	
More than 1x/week	16(39%)	46(11.6%)	
*PSQI – medicines*			0.01^*^
Less than 1x/week	38(92.7%)	390(98.5%)	
1x/week or more	3(7.3%)	6(2.5%)	
*PSQI – daytime dysfunctions*			<0.001^*^
Less than 1x/week	25(61%)	344(86.9%)	
1x/week or more	16(39%)	52(13.1%)	

PSQI and domains	Sex		*p*-value
	Feminine	Masculine	
*PSQI – total*			<0.001^*^
Good sleep quality	54 (24.3%)	101 (47%)	
Bad sleep quality	168 (75.7%)	114 (53%)	
*PSQI – subjective*			<0.001^*^
Good/very good	85 (38.3%)	41 (19.1%)	
Bad/Very bad	137 (61.7%)	174 (80.9%)	
*PSQI – latency*			<0.001^*^
>15 min – 30 min	146 (65.8%)	170 (79.1%)	
Above 30 min	76 (34.2%)	45 (20.9%)	
*PSQI – duration*			<0.001^*^
6 h or more	193 (86.9%)	203 (94.4%)	
Less than 6 h	29 (13.1%)	12 (5.6%)	
*PSQI – disorders*			<0.001^*^
*1x/week or less*	167 (75.2%)	208 (96.7%)	
*More than 1x/week*	55 (24.8%)	7 (3.3%)	
*PSQI – medication*			0.01^*^
*Less than 1x/week*	221 (99.5%)	207 (96.3%)	
*1x/week or more*	1 (0.5%)	8 (3.7%)	
*PSQI – Daytime dysfunctions*			0.04^*^
*Less than 1x/week*	180 (81.1%)	189 (87.9%)	
*1x/week or more*	42 (18.9%)	26 (12.1%)	

*p < 0.05; %, relative frequency/percentage; PSQI, Pittsburgh Sleep Quality Index.

Data regarding time in minutes of physical activity were correlated with the total PSQI index and the sleep latency domain. Weak negative correlations were observed between weekly physical activity time and both the total PSQI score (*r* = −0.104; *p* = 0.04) and sleep latency (*r* = −0.102; *p* = 0.03), indicating that higher levels of physical activity were associated with slightly better sleep parameters (Table 4).

**Table 4:** Correlations between the time in minutes exercising and the total PSQI value.

Variable	Value	*p*
PSQI – total		
Time in minutes spent exercising	−0.104	0.04*
PSQI – latency		
Time in minutes spent exercising	−0.102	0.03*

PSQI, Pittsburgh Sleep Quality Index; *p* < 0.05 indicates statistical significance.

Crude analysis suggested an association between physical activity and sleep quality; however, this association was no longer significant after adjustment for sex and age. In the adjusted logistic regression analysis, physical activity was not significantly associated with sleep quality (OR 1.49; 95% CI 0.95–2.33; *p* = 0.079). Sex remained significantly associated with sleep quality, while age was not significantly associated (Table 5).

**Table 5:** Crude and adjusted associations between physical activity and sleep quality.

Variable	OR (95% CI)	*p*-value	Adjusted OR (95% CI)*	*p*-value
Physical activity (min/week)	-	-	0.999 (0.998–1.000)	0.007
Physical activity (≥150 min/week)	1.87 (1.16–3.43)	0.012	1.49 (0.95–2.33)	0.079
Sex (female vs. male)	-	-	2.81 (1.86–4.24)	<0.001
Age (years)	-	-	1.09 (0.94–1.25)	0.249

OR, odds ratio; CI, confidence interval. Adjusted for sex and age. Reference category for physical activity: <150 min/week. < Less than; * Indicates statistical significance; > Greater than.

**Figure 1:**
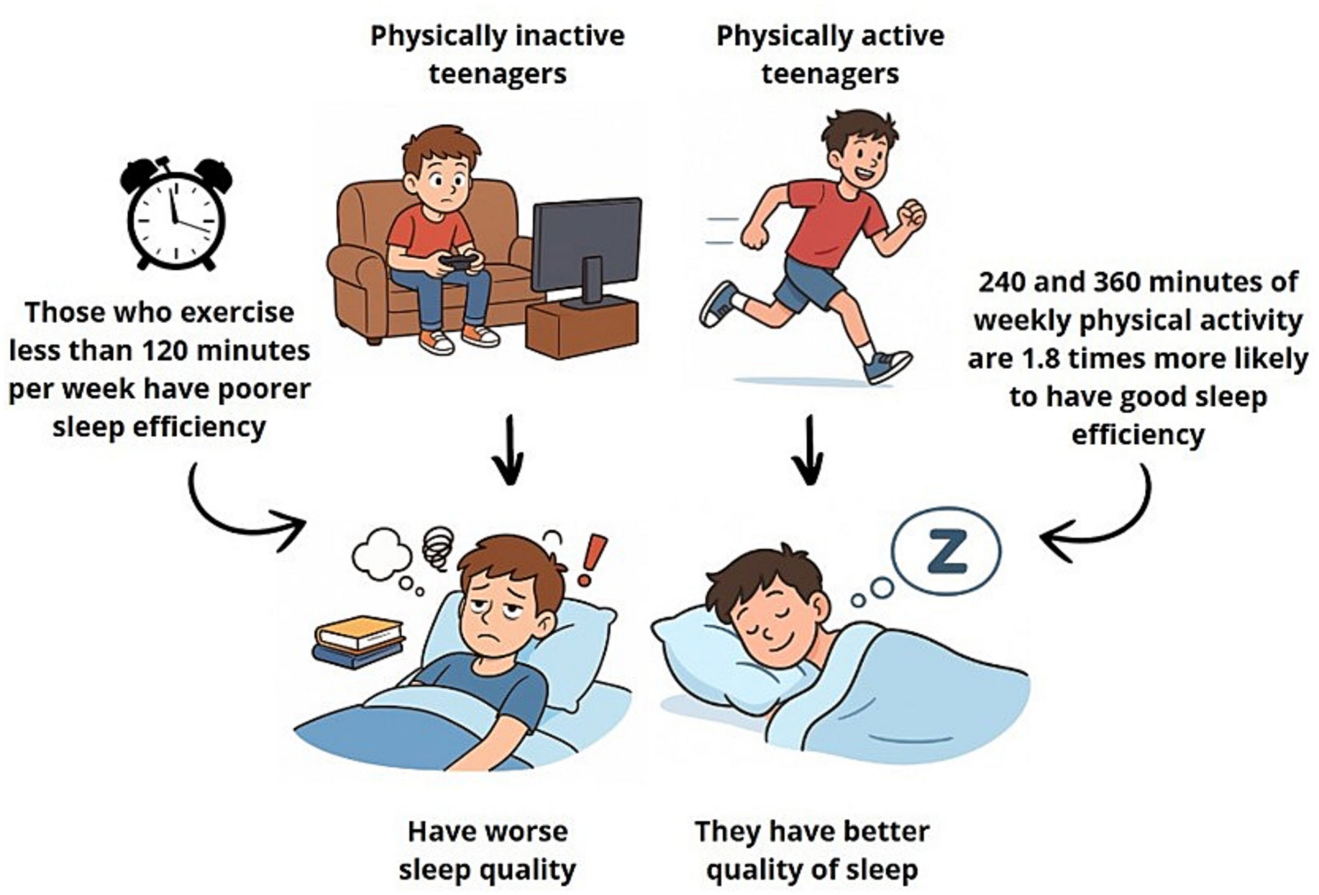
Relationship between sedentary and healthy habits, highlighting their influence on sleep quality.

Exploratory analyses using physical activity quartiles suggested a potential dose–response relationship with sleep efficiency; however, these findings should be interpreted with caution. Figure 1 shows the association between physical activity levels and sleep quality in adolescents.

## 4 Discussion

The present study aimed to evaluate sleep quality in Italian adolescents and to ex-amine its associations with self-perceived academic performance and physical activity. The findings showed that only a minority of participants reported good or excellent subjective sleep quality (36%), despite the majority reporting good or excellent academic performance and being classified as physically active. In contrast, the PSQI results indicated that most adolescents presented poor sleep quality, highlighting a discrepancy between self-perceived sleep quality and assessment using a validated multidimensional instrument.

Previous studies have shown that inadequate sleep is common during adolescence and is associated with negative physical and psychological outcomes, including impaired mental health and reduced academic functioning (1, 35, 36). However, due to the cross-sectional design of the present study, the observed relationships should be interpreted as associations rather than causal effects. Poor sleep quality has been associated with deficits in attention, memory, emotional regulation, and daytime functioning, which may negatively affect adolescents’ academic experiences and daily life (37).

In the present study, self-reported academic performance was associated with subjective sleep disturbance, use of sleep medication, and daytime dysfunction domains of the PSQI. Although most participants reported good or excellent academic performance, it is important to acknowledge that academic performance was assessed using a single, non-validated self-report question, which limits the strength of interpretation of these findings. Nevertheless, these results are consistent with previous literature indicating that insufficient or poor-quality sleep is associated with cognitive and behavioral difficulties in adolescents (36, 37).

Regarding physical activity participation, adolescents in the sample reported a relatively high weekly amount of time dedicated to practice. However, this finding should be interpreted with caution, since the measure was based on general physical activity per week and did not assess total physical activity in all domains of daily life, nor the intensity of the activity. Therefore, the results should not be interpreted as direct evidence of adherence to World Health Organization physical activity recommendations. Additionally, the use of self-reported measures may have introduced measurement error, potentially attenuating observed associations.

Sedentary behavior and increased screen time during the COVID-19 lockdown have been associated with poorer sleep outcomes in adolescents (38–40). These contextual factors may have influenced both sleep patterns and physical activity levels in the present sample and should be considered when interpreting the results.

In the present analyses, physical activity showed small but statistically significant correlations with sleep quality outcomes. In the adjusted regression model, when physical activity was analyzed as a continuous variable, higher levels of physical activity were significantly associated with lower odds of poor sleep quality. However, when physical activity was categorized according to WHO recommendations (≥150 min/week), the association was no longer statistically significant. This discrepancy suggests that the relationship between physical activity and sleep quality may be better characterized as a continuous or dose–response association rather than a threshold-based effect.

The attenuation of the association between physical activity and sleep quality observed in the categorical model highlights the potential influence of analytical decisions on study findings. While no significant association was observed using a threshold-based approach (≥150 min/week), the continuous model revealed a significant association, suggesting that the relationship between physical activity and sleep quality may be better characterized as dose–response rather than dichotomous.

This finding indicates that categorization may obscure meaningful associations and reinforces the importance of preserving continuous data when possible.

Although the magnitude of the associations was small, this finding is consistent with previous literature indicating that behavioral factors such as physical activity often exert modest but meaningful effects on sleep outcomes. When interpreted cumulatively, increases in physical activity may result in clinically relevant improvements in sleep quality.

Exploratory analyses using physical activity quartiles further supported a potential dose–response relationship, reinforcing the findings observed in the continuous model. However, these results should be interpreted with caution due to their exploratory nature and the cross-sectional design.

Sex remained a strong independent predictor of sleep quality. This finding is consistent with previous literature showing that female adolescents are more likely to report poorer sleep quality, which may be related to hormonal fluctuations, higher prevalence of internalizing symptoms such as anxiety, and differences in sleep hygiene behaviors. Psychosocial factors, including stress and academic pressure, may also contribute to these differences.

Overall, the findings suggest that during the COVID-19 pandemic, Italian adolescents experienced poor sleep quality despite reporting relatively high levels of physical activity and positive perceptions of academic performance. These results corroborate recent evidence suggesting that movement behaviors throughout the 24-h day, including physical activity, sedentary behavior, and sleep, are interdependent and jointly influence cognitive and academic outcomes (34).

Importantly, the observed association between physical activity and sleep quality appears to follow a dose–response pattern, rather than a simple dichotomous relationship. This highlights the complexity of sleep–behavior interactions and reinforces the importance of analytical approaches that preserve the continuous nature of behavioral variables.

Importantly, the present findings highlight how analytical decisions can influence the interpretation of results. The use of a continuous approach to physical activity allowed the identification of a significant association that was not detected when using a categorical threshold. This reinforces concerns raised in the literature regarding the loss of information and statistical power when continuous variables are dichotomized.

These findings emphasize the importance of carefully selecting analytical strategies when investigating behavioral determinants of health outcomes in observational studies.

## 5 Conclusion

The present study demonstrated a high prevalence of poor sleep quality among Italian adolescents during the COVID-19 pandemic. Although physical activity was weakly correlated with some sleep parameters, it was not independently associated with sleep quality after adjustment for sex and age. These findings highlight the complexity of sleep-related behaviors in adolescents and the importance of considering confounding factors when interpreting associations in observational studies.

### 5.1 Limitations and future studies

The current study presents some limitations, such as the cross-sectional design of the study, which does not allow causal inferences from the data found. Cross-sectional measurement designs are biased towards establishing the temporal order between variables. Therefore, we cannot draw conclusions about the causal relationships between the variables investigated. Longitudinal studies are needed to clarify this aspect. Furthermore, the data were self-reported, which can cause memory bias and response bias, for example, due to social desirability, recall period, and/or selective recall (41). As it is composed only of adolescents from one region of Italy, the population of the present study may be influenced by a local academic and cultural system, and caution is required when extrapolating the results.

Third, although an adjusted regression model was performed, including sex and age as covariates, residual confounding cannot be ruled out. Other relevant factors, such as body mass index, mental health status, screen time, and socioeconomic conditions, were not included in the adjusted model and may have influenced sleep quality.

Furthermore, physical activity was analyzed using different approaches (continuous, categorical, and quartiles), which may increase the risk of type I error due to multiple comparisons. Although these strategies were used to explore different aspects of the data, the findings should be interpreted with caution. Another parameter is the self-perception of minutes of physical activity per week, which can lead the participant to inaccurate answers. We recognize that in the present study, an instrument for analyzing physical activity or exercise would be more appropriate, especially due to the age of the participants, which could be classified as more specific minutes per week of physical activities.

Finally, the sample was composed of adolescents from a single region of Italy, which may limit the generalizability of the results to other populations with different cultural and educational contexts.

Future research is needed to reduce biases associated with the use of subjective data. We suggest using objective sleep duration and quality measurement instruments, such as actigraphy and polysomnography, to better understand the long-term implications of adolescent sleep. Furthermore, based on our results for sleep quality, other sleep constructs, such as wake-up time and chronotype, should be investigated and controlled through sleep-related tests, as well as the effects of school start times. Correlations between elementary and high school start times and students’ sleep duration, for example, appear to be closely associated with sleep and may have a significant effect on academic performance (5).

### 5.2 Strengths, innovations, and applications

The present study evaluated sleep quality and physical activity in a relatively large sample of Italian adolescents, contributing to the literature by integrating multiple dimensions of sleep assessed through the PSQI, including efficiency, latency, and daytime dysfunction. Another strength of this study is the use of different analytical approaches, including correlation analyses and adjusted regression models, allowing a more comprehensive examination of the relationship between physical activity and sleep outcomes. Importantly, the inclusion of adjusted analyses improved the robustness of the findings by accounting for potential confounding factors. The results highlight the complexity of the relationship between physical activity and sleep quality in adolescents, suggesting that observed associations may be influenced by other variables, such as sex. These findings may be relevant for researchers and professionals in health and education, emphasizing the need for multifactorial approaches when addressing sleep health in adolescents.

## Data Availability

The raw data supporting the conclusions of this article will be made available by the authors, without undue reservation.

## Author’s note

All authors confirm that this manuscript is a transparent and honest account of the research reported. This research is related to a previous study by the same authors, entitled “Mood states of active and insufficiently active adolescents related to sleep quality, gender, academic performance, and guidelines for the post-COVID-19 scenario.” The previous study was conducted on October 31, 2024, and this submission focuses on February 17, 2026. The study follows the methodology explained at https://www.frontiersin.org/journals/psychology/articles/10.3389/fpsyg.2024.1494456/full.
