# Supplementary material for: Sleep quality, physical activity, and perceived academic performance of Italian adolescents
Source: Front Public Health. 2026 May 13;14:1810897. doi: 10.3389/fpubh.2026.1810897 (PMC13212458; doi:10.3389/fpubh.2026.1810897)
Supplement: Supplementary file 1 [file Data_Sheet_1.DOCX]

**Supplementary Material**

**Figure S1 -** Histogram of Total Pittsburgh Index, exercise time (minutes), and age


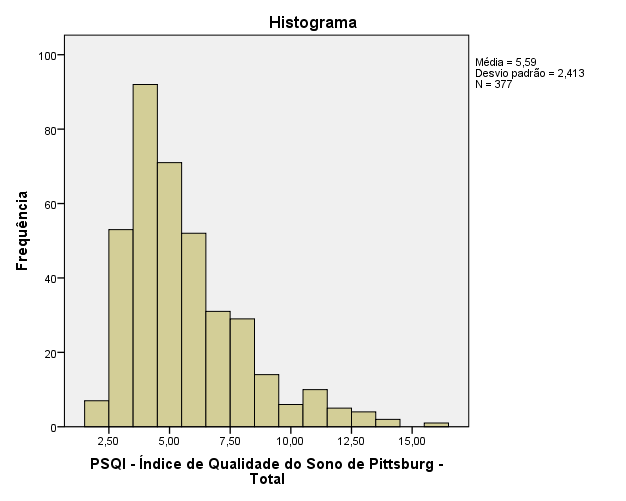

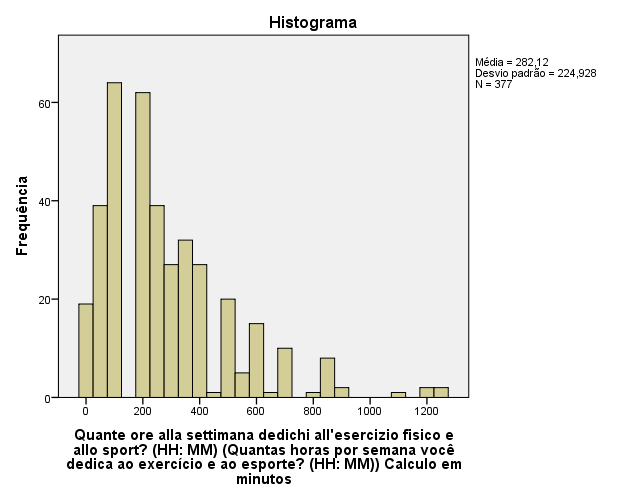

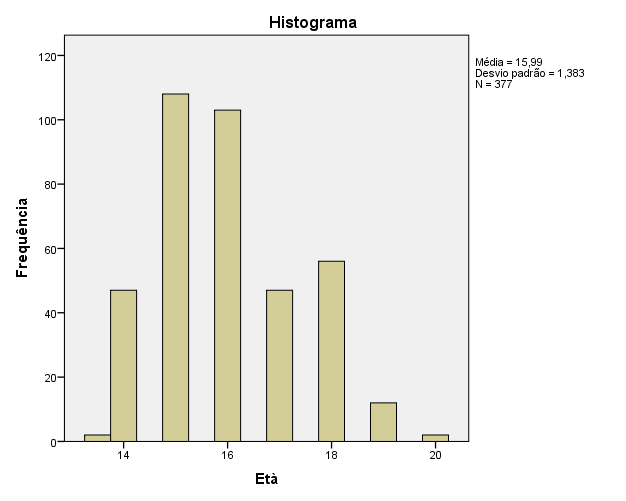


**Figure S2** – Q–Q plots assessing normality of main continuous variables Total Pittsburg Index, exercise time (minutes) and age


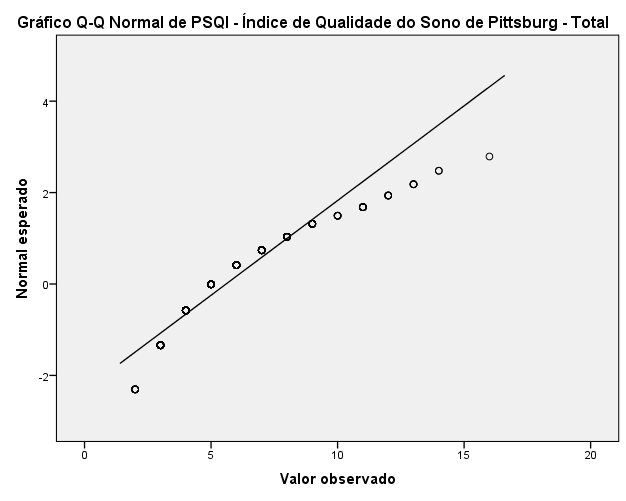

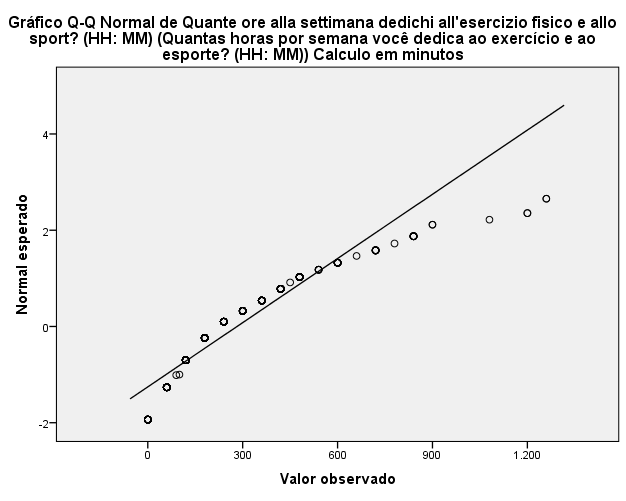

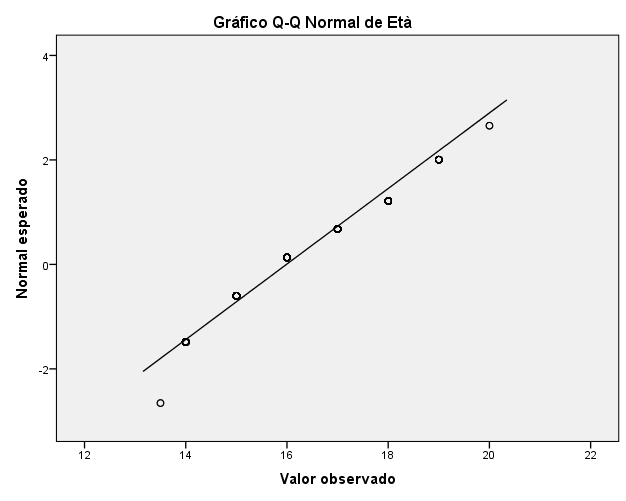


**Figure S3 -** Linearity diagnostic: Association between self-reported physical activity levels and PSQI total score, including the linear regression trend line and coefficient of determination

**
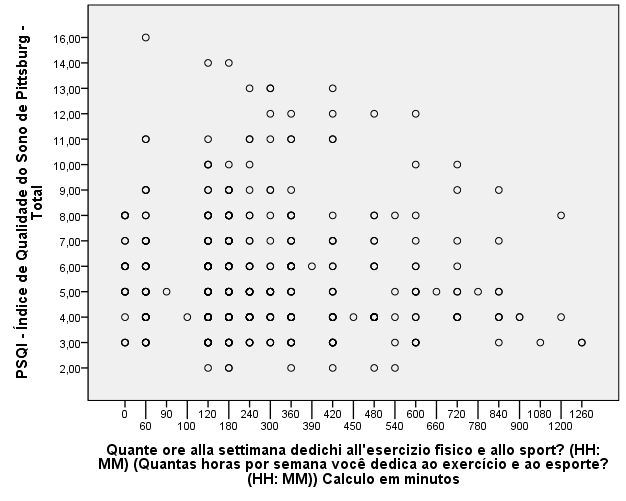

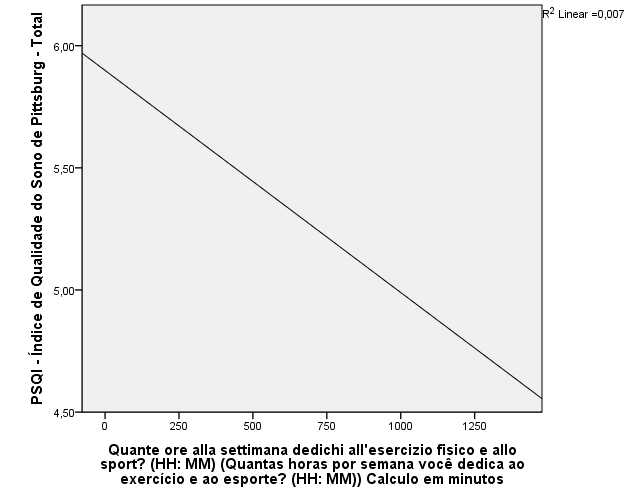
**

**Table S1** – Tests of Normality

| **Tests of Normality** | | | | | | |
| --- | --- | --- | --- | --- | --- | --- |
|  | Kolmogorov-Smirnov^a^ | | | Shapiro-Wilk | | |
|  | Estatística | df | Sig. | Estatística | df | Sig. |
| Età | ,184 | 388 | ,000 | ,922 | 388 | ,000 |
| Quante ore alla settimana dedichi all'esercizio fisico e allo sport? (HH: MM) (Quantas horas por semana você dedica ao exercício e ao esporte? (HH: MM)) Calculo em minutos | ,164 | 388 | ,000 | ,874 | 388 | ,000 |
| PSQI - Índice de Qualidade do Sono de Pittsburg - Total | ,179 | 388 | ,000 | ,892 | 388 | ,000 |
| a. Lilliefors Significance Correction | | | | | | |

**Table S2** - VIF e Tolerance

| **Coeficientes^a^** | | | | | | | | |
| --- | --- | --- | --- | --- | --- | --- | --- | --- |
| Modelo | | Coeficientes não padronizados | | Coeficientes padronizados | t | Sig. | Estatísticas de colinearidade | |
|  |  | B | Modelo padrão | Beta |  |  | Tolerância | VIF |
| 1 | (Constante) | 4,769 | 1,392 |  | 3,426 | ,001 |  |  |
|  | Età | ,101 | ,086 | ,058 | 1,176 | ,240 | ,994 | 1,006 |
|  | Sesso | -1,198 | ,238 | -,250 | -5,042 | ,000 | ,983 | 1,017 |
|  | Quante ore alla settimana dedichi all'esercizio fisico e allo sport? (HH: MM) (Quantas horas por semana você dedica ao exercício e ao esporte? (HH: MM)) Calculo em minutos | -,001 | ,001 | -,052 | -1,048 | ,295 | ,980 | 1,020 |
| a. Variável dependente: PSQI - Índice de Qualidade do Sono de Pittsburg - Total | | | | | | | | |

**Table S3** - Associations between study variables and PSQI domains with Benjamini–Hochberg FDR-adjusted p-values

| **Comparison** | **p-value (original)** | **p-value (FDR adjusted)** |
| --- | --- | --- |
| Physical activity vs PSQI Total | 0.030 | 0.033 |
| Academic Performance vs PSQI – Subjective | 0.020 | 0.024 |
| Academic Performance vs PSQI – Disorders | <0.001 | 0.012 |
| Academic Performance vs PSQI – Medicines | 0.010 | 0.015 |
| Academic Performance vs PSQI – Daytime dysfunctions | <0.001 | 0.006 |
| Sex vs PSQI – Total | <0.001 | 0.004 |
| Sex vs PSQI – Subjective | <0.001 | 0.003 |
| Sex vs PSQI – Latency | <0.001 | 0.002 |
| Sex vs PSQI – Duration | <0.001 | 0.002 |
| Sex vs PSQI – Disorders | <0.001 | 0.002 |
| Sex vs PSQI – Medication | 0.010 | 0.013 |
| Sex vs PSQI – Daytime Dysfunctions | 0.040 | 0.040 |

*Benjamini–Hochberg false discovery rate (FDR) correction was applied to control for multiple comparisons.
All p-values < 0.05 were considered statistically significant.

**Table S4 -** Associations between PSQI domains and exercise practice, academic performance, and sex.

| **PSQI and domains** | **Exercise practice** | | **P value** |
| --- | --- | --- | --- |
|  | Insufficiently active | Active |  |
| *PSQI Total*  Good sleep quality  Poor sleep quality | 41(26.5%)  99(35.1%) | 114(73.5%)  183(64.9%) | 0.03* |
| **PSQI and domains** | **Academic Performance** | | **P value** |
|  | Insufficiently/mediocre | Normal/excellent |  |
| *PSQI – Subjective*  Good/very good  Bad/Very bad | 18(43.9%)  23(56.1%) | 108(27.3%)  288(72.7%) | 0.02* |
| *PSQI – Disorders*  1x/week or less  More than 1x/week | 25(61%)  16(39%) | 350(88.4%)  46(11.6%) | < 0,001* |
| *PSQI – Medicines*  Less than 1x/week  1x/week or more | 38(92.7%)  3(7.3%) | 390(98.5%)  6(2.5%) | 0.01* |
| *PSQI – Daytime dysfunctions*  Less than 1x/week  1x/week or more | 25(61%)  16(39%) | 344(86.9%)  52(13.1%) | < 0,001* |

| **PSQI and domains** | **Sex** | | **P value** |
| --- | --- | --- | --- |
|  | Feminine | Masculine |  |
| *PSQI – Total*  Good sleep quality  Bad sleep quality | 54 (24.3%)  168 (75.7%) | 101 (47%)  114 (53%) | < 0,001* |
| *PSQI – Subjective*  Good/very good  Bad/Very bad | 85 (38.3%)  137 (61.7%) | 41 (19.1%)  174 (80.9%) | < 0,001* |
| *PSQI – Latency*  >15 min – 30 min  Above 30 min | 146 (65.8%)  76 (34.2%) | 170 (79.1%)  45 (20.9%) | < 0,001* |
| *PSQI – Duration*  6 hours or more  Less than 6 hours | 193 (86.9%)  29 (13.1%) | 203 (94.4%)  12 (5.6%) | < 0,001* |
| *PSQI – Disorders*  *1x/week or less*  *More than 1x/week* | 167 (75.2%)  55 (24.8%) | 208 (96.7%)  7 (3.3%) | < 0,001* |
| *PSQI – Medication*  *Less than 1x/week*  *1x/week or more* | 221 (99.5%)  1 (0.5%) | 207 (96.3%)  8 (3.7%) | 0.01* |
| *PSQI – Daytime Dysfunctions*  *Less than 1x/week*  *1x/week or more* | 180 (81.1%)  42 (18.9%) | 189 (87.9%)  26 (12.1%) | 0.04* |

*p<0.05; %=relative frequency/percentage; PSQI = Pittsburgh Sleep Quality Index;

**Table S5** – Logistic regression model with physical activity as a continuous variable

| **Variable** | **B** | **SE** | **OR (Exp(B))** | **95% CI** | **p-value** |
| --- | --- | --- | --- | --- | --- |
| Age (years) | — | — | 1.09 | 0.93 – 1.27 | 0.284 |
| Sex (ref: female)* | — | — | 0.39 | 0.26 – 0.58 | <0.001 |
| Physical activity (min/week) | — | — | 0.999 | 0.998 – 1.000 | 0.007 |

Legend: OR = Odds Ratio; CI = Confidence Interval; *Reference category should be specified (e.g., female = 0, male = 1); Logistic regression adjusted for age and sex; Significance level set at p < 0.05

**Model fit:** Hosmer–Lemeshow: p = 0.365; Nagelkerke R² = 0.10

**Table S6** – Logistic regression model with physical activity categorized according to WHO recommendations

| **Variable** | **B** | **SE** | **OR (Exp(B))** | **95% CI** | **p-value** |
| --- | --- | --- | --- | --- | --- |
| Age (years) | — | — | 1.09 | 0.94 – 1.26 | 0.249 |
| Sex (ref: female)* | — | — | 0.35 | 0.24 – 0.54 | <0.001 |
| Physical activity (<150 vs ≥150 min/week) | — | — | 1.49 | 0.95 – 2.33 | 0.079 |

Legend: OR = Odds Ratio; CI = Confidence Interval; *Reference category should be specified (e.g., female = 0, male = 1); Logistic regression adjusted for age and sex; Significance level set at p < 0.05

**Model fit:** Hosmer–Lemeshow: p = 0.452; Nagelkerke R² = 0.092

**Table S7** – Assessment of linearity in the logit using the Box–Tidwell procedure

| **Variable** | **B** | **SE** | **OR (Exp(B))** | **95% CI** | **p-value** |
| --- | --- | --- | --- | --- | --- |
| Age (years) | — | — | 1.09 | 0.93 – 1.27 | 0.283 |
| Sex (ref: female)* | — | — | 0.39 | 0.26 – 0.58 | <0.001 |
| Physical activity (min/week) | — | — | 0.995 | — | 0.452 |
| PA × ln(PA) (interaction term) | — | — | — | — | 0.571 |

Legend: OR = Odds Ratio; CI = Confidence Interval; *Reference category should be specified (e.g., female = 0, male = 1); Logistic regression adjusted for age and sex; Significance level set at p < 0.05
